# Interactive Effects of [CO_2_] and Temperature on Plant Chemistry of Transgenic *Bt* Rice and Population Dynamics of a Non-Target Planthopper, *Nilaparvata lugens* (Stål) under Different Levels of Soil Nitrogen

**DOI:** 10.3390/toxins11050261

**Published:** 2019-05-08

**Authors:** Yanmin Liu, Zhihao Dang, Yanhui Wang, Megha N. Parajulee, Fajun Chen

**Affiliations:** 1 Department of Entomology, College of Plant Protection, Nanjing Agricultural University, Nanjing 210095, China; 2017202044@njau.edu.cn (Y.L.); dangqwe126@163.com (Z.D.); 2018102061@njau.edu.cn (Y.W.); 2 Zhengzhou Customs, Zhengzhou 410003, China; 3 Texas A&M AgriLife Research and Extension Center, Lubbock, TX 79403, USA; mparajul@ag.tamu.edu

**Keywords:** elevated CO_2_, high temperature, fertilizer-N level, transgenic *Bt* rice, plant chemistry, rice planthopper, population dynamics

## Abstract

Gaining a better understanding of the interactive effect of projected atmospheric CO_2_ level increase and the Earth’s rising temperature on plant chemistry (nutritional and defensive characteristics) of transgenic crops is essential when attempting to forecast the responses of target and non-target insects to climate change. In this study, effects of carbon dioxide (CO_2_; elevated versus ambient), temperature (T; high versus low), and their interactions on leaf nitrogen content (N%) and C:N ratio of transgenic *Bt* rice and its non-*Bt* isoline grown under low- and high-N fertilizer were systematically analyzed together with the resulting insect population dynamics of a non-target planthopper *Nilaparvata lugens* (Stâl) in open-top-chamber experiments. The results indicated that under low-N treatment, elevated CO_2_ at low T (i.e., eCO_2_) (compared to ambient CO_2_ at low T, i.e., CK) significantly decreased N% and *Bt*-toxin content and significantly increased C:N ratio in leaf sheath and leaf of *Bt* rice, especially during the tillering stage, whereas inverse effects of high T were shown on the plant chemistry of *Bt* rice, especially during heading stage. The combination of elevated CO_2_ and high T (i.e., Combined) (in contrast to CK) significantly increased N% and decreased C:N ratio in leaf sheath of *Bt* rice during the heading stage under low-N fertilizer, while significantly decreased N% and increased C:N ratio in leaf of *Bt* rice during the tillering stage, regardless of fertilizer-N level, and significantly increased *Bt*-toxin content in leaf sheath and leaf during the tillering stage under both low- and high-N. Moreover, no discernable relationships between *Bt*-toxin content and N% or leaf C:N ratio were observed at any CO_2_ or N levels evaluated. Furthermore, transgenic treatment, temperature and fertilizer-N level interactions, and CO_2_ and fertilizer-N level interactions all significantly affected the population dynamics of *N. lugens*. Specifically, high-N significantly enhanced the population dynamics of *N. lugens* fed on non-*Bt* rice grown under eTemp and *Bt* cultivar significantly reduced the population dynamics of *N. lugens* under eCO_2_ regardless of N fertilizer levels. The study demonstrates that the planting of transgenic *Bt* rice would not increase the risk of increased *N. lugens* severity under the combined condition of elevated CO_2_ and increased temperature, particularly under moderate level of N fertility.

## 1. Introduction

To date, many transgenic *Bt* crops (ab., *Bt* crops; e.g. soybeans and cotton) have been planted worldwide and shown to offer resistance to specific target pests, mainly chewing insects [1,2]. *Bt* crops have thus far been used to manage a wide spectrum of insect pests, including *Helicoverpa armigera* Hübner, *Helicoverpa zea*, *Chilo suppressalis* (Walker), *Heliothis virescens*, *Empoasca fabae* Harris, and *Aphis gossypii* Glover [3,4,5,6]. On November 27, 2009, China’s Ministry of Agriculture (MOA) issued biosafety certificates for transgenic *Bt* rice (ab., *Bt* rice) expressing fused *Cry1Ab/Ac* genes (cv. Huahui-1 and *Bt* Shanyou-63). These two *Bt* rice lines were issued biosafety certificates for the laboratory and field evaluation in 2015. Laboratory and field tests have shown that these two lines of *Bt* rice exhibited high resistance to the target lepidoptera pests [7,8].

The brown planthopper *N. lugens* mainly feeds on rice plants and sucks phloem saps, and outbreaks of it are frequent. It shows no sensitivity to *Bt* toxin produced by *Bt* rice and may even thrive on Bt rice under elevated CO_2_ and rising temperature conditions [9]. Compared with that fed on non-*Bt* rice, *N. lugens* showed no difference in nymphal development or total development time from egg to adult, or in their survival rate when fed on *Bt* rice, nor did they show any preference for one or the other rice cultivar [10,11]. However, Chen et al. found that *Bt* rice (cv., KMD expressing *Cry1Ab*) delayed nymphal development, decreased female fecundity, and reduced population density of *N. lugens* [11]. Its close relative, the white-backed planthopper, *Sogatella furcifera* Horváth, on the other hand, thrived on *Bt* rice under elevated CO_2_ [12]. To what extent *Bt* rice can control rice planthoppers is largely unclear. Hence, the planthopper is an ideal pest insect for investigation into the potential effects of rising temperature in conjunction with elevated CO_2_ on insect or host–pest interaction in the coming decades.

Previous studies have examined the effect of CO_2_ and rising ambient temperature singly or in combination with two variables on plant growth [13,14,15,16], insect pests [17,18,19], and plant–herbivore interactions [20]. While overabundant CO_2_ tends to promote plant photosynthesis [20] and increase biomass [21], it also causes a reduction in foliar nitrogen content (N%) and an increase in C:N ratio in most plants, especially C_3_ plants [22,23], resulting in an increased consumption rate in insects to overcome the detrimental lack of nutrition. While a higher temperature generally shortens the growth period of plants and increases above-ground biomass and nitrogen content [24], it is also directly responsible for faster development and earlier maturity of insect herbivores by increasing their metabolic rates [25]. Globally rising temperatures also make it possible for overwintering insects to extend their winter range and establish year-round populations [25]. Hu et al. reported the *N. lugens* has developed into a major pest during the past ten years because of rising temperatures [26]. Insect pests may be affected not only by the nutrient balance of their host plants, such as decreased nitrogen and increased C-based secondary metabolites under global climate change [27,28], but also by changes in surface waxes, trichomes, toughness, and leaf microstructure of plants [29]. These plant physical parameters may also need to be taken into consideration as we examine the quantities of foliar nitrogen, C:N ratios, *Bt* toxin content, and insect pests’ abundances.

Therefore, our research questions are (a) whether or not the planting of *Bt* rice is likely to cause a catastrophic explosion of brown planthopper populations as global temperatures and CO_2_ levels are bound to rise, (b) whether the production of *Bt* toxin will drain the plants’ energy away from the accumulation of nitrogen and thus diminish the nutritional quality of the rice crop, and (c) whether an ever greater overabundance of CO_2_ will lead to a higher nitrogen demand and slow down *Bt* toxin production to the point where the resistance of *Bt* rice plants to non-target insect pests could be seriously impaired.

## 2. Results

### 2.1. N Content (N%) and C:N Ratio of Bt and Non-Bt Rice Cultivars Influenced by CO_2_, T, and Nitrogen Fertility Levels

**Figure 1:**
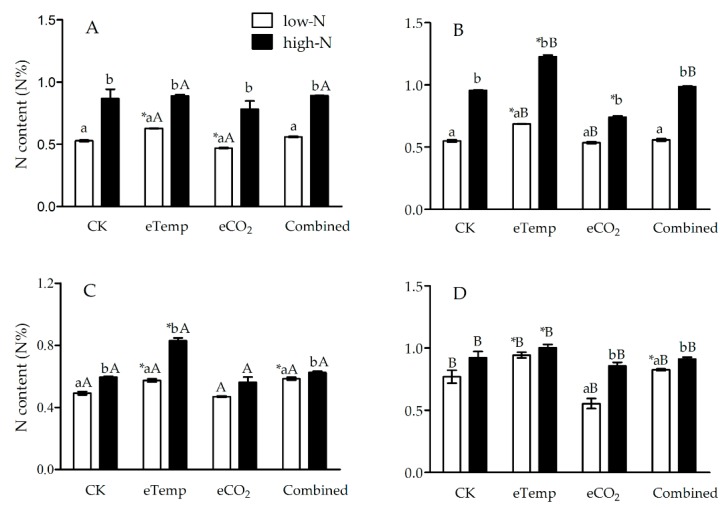
The nitrogen content (N%) in leaf sheath of transgenic *Bt* rice (cv. HH1; **A**, **C**) and its parental isoline of non-*Bt* rice (cv. MH63; **B**, **D**) during the tillering (**A**, **B**) and heading (**C**, **D**) stages, grown with low- and high-N fertilizer under various CO_2_ and temperature (T) combination in open-top chambers. (**Note:** HH1—*Bt* rice expressing *cry1Ab*/*Ac* genes; MH63—the parental isoline of HH1; CK—ambient CO_2_ and low-T; eTemp—ambient CO_2_ and high-T; eCO_2_—elevated CO_2_ and low-T; Combined—elevated CO_2_ and high-T; *, the lowercase (a, b) and uppercase letters (A, B) indicated significant difference in N% of *Bt* rice or non-*Bt* rice under eTemp, eCO_2_ or Combined treatment compared with CK at same fertilizer-N level, and between low-N and high-N for *Bt* rice or non-*Bt* rice grown under same levels of CO_2_ and T, and between *Bt* rice and non-*Bt* rice grown under same levels of CO_2_, T and N fertilizer by the Turkey-test at *p* < 0.05, respectively.

**Figure 2:**
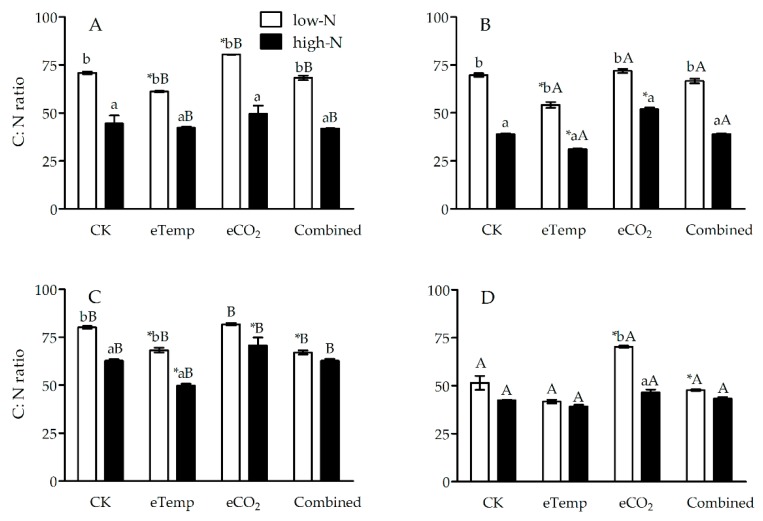
The C:N ratio in leaf sheath of transgenic *Bt* rice (cv. HH1; **A**, **C**) and its parental isoline of non-*Bt* rice (cv. MH63; **B**, **D**) during the tillering (**A**, **B**) and heading (**C**, **D**) stages, grown with low- and high-N fertilizer under two levels of CO_2_ and temperature (T) in open-top chambers. (Note: *, the lowercase (a, b) and uppercase letters (A, B) indicated significant difference in N% of *Bt* rice or non-*Bt* rice under eTemp, eCO_2_ or Combined treatment compared with CK at same fertilizer-N level, and between low-N and high-N for *Bt* rice or non-*Bt* rice grown under same levels of CO_2_ and T, and between *Bt* rice and non-*Bt* rice grown under same levels of CO_2_, T and N fertilizer by the Turkey-test at *p* < 0.05, respectively).

**Figure 3:**
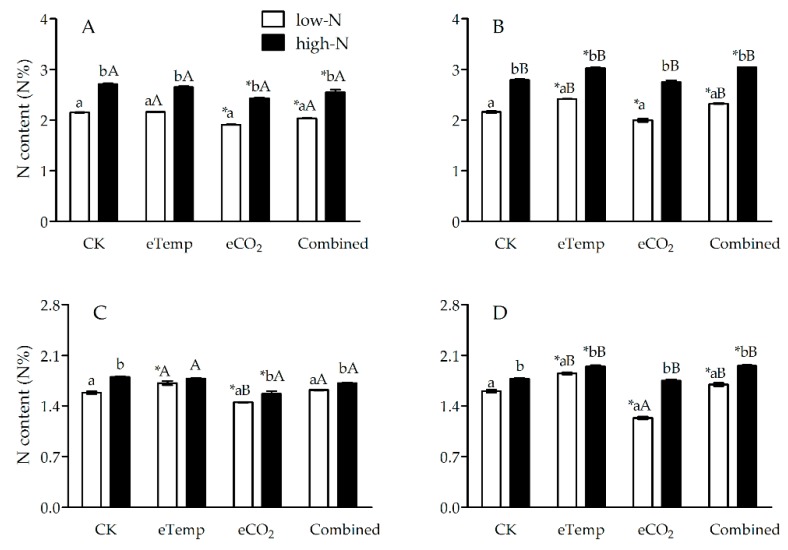
The nitrogen content (N%) in leaf of transgenic *Bt* rice (cv. HH1; **A**, **C**) and its parental isoline of non-*Bt* rice (cv. MH63; **B**, **D**) during the tillering (**A**, **B**) and heading (**C**, **D**) stages, grown with low- and high-N fertilizer under various CO_2_ and temperature (T) combination in open-top chambers. (Note: *, the lowercase (a, b) and uppercase letters (A, B) indicated significant difference in N% of *Bt* rice or non-*Bt* rice under eTemp, eCO_2_ or Combined treatment compared with CK at same fertilizer-N level, and between low-N and high-N for *Bt* rice or non-*Bt* rice grown under same levels of CO_2_ and T, and between *Bt* rice and non-*Bt* rice grown under same levels of CO_2_, T and N fertilizer by the Turkey-test at *p* < 0.05, respectively).

**Figure 4:**
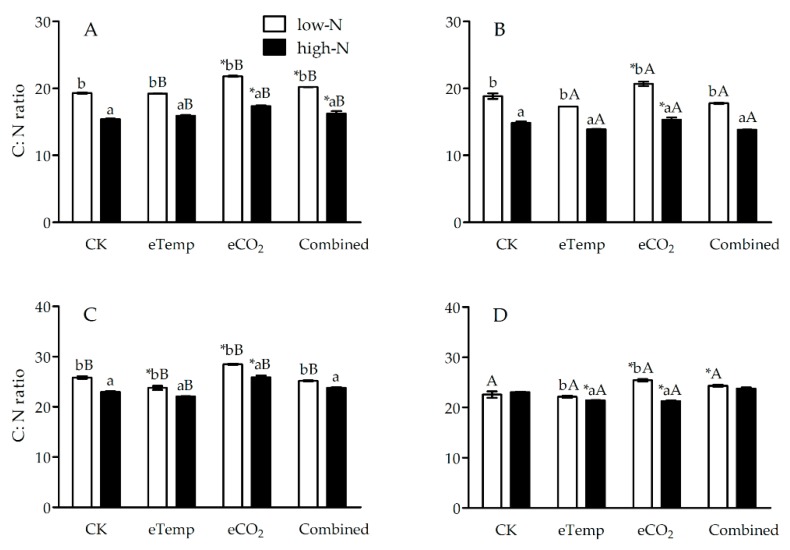
The C:N ratio in leaf of transgenic *Bt* rice (cv. HH1; **A**, **C**) and its parental isoline of non-*Bt* rice (cv. MH63; **B**, **D**) during the tillering (**A**, **B**) and heading (**C**, **D**) stages, grown with low- and high-N fertilizer under various CO_2_ and temperature (T) combination in open-top chambers. (Note: *, the lowercase (a, b) and uppercase letters (A, B) indicated significant difference in N% of *Bt* rice or non-*Bt* rice under eTemp, eCO_2_ or Combined treatment compared with CK at same fertilizer-N level, and between low-N and high-N for *Bt* rice or non-*Bt* rice grown under same levels of CO_2_ and T, and between *Bt* rice and non-*Bt* rice grown under same levels of CO_2_, T and N fertilizer by the Turkey-test at *p* < 0.05, respectively).

Elevated CO_2_ (compared to ambient CO_2_) and *Bt* cultivar (in contrast to non-*Bt* rice) both decreased N% and increased C:N ratio in leaf sheath and leaf during both the tillering and heading stages (Figure 1, Figure 2, Figure 3 and Figure 4), while inverse effects of high T (compared to low T) and high-N fertilizer (compared to low-N fertilizer) and neutral effects of elevated CO_2_ and high T (i.e., combination effects) were observed compared with CK treatment (Figure 1, Figure 2, Figure 3 and Figure 4).

#### 2.1.1. N% and C:N Ratio in Leaf Sheath

At low T, CO_2_, fertilizer-N level, and transgenic treatment all significantly affected N% and C:N ratio in leaf sheath during the tillering and heading stages (*p* < 0.001 or 0.01), except for the effect of transgenic treatment on N% during the tillering stage (Table 1 and Table 2). A significant interaction was observed between CO_2_ and fertilizer-N levels on N% during the tillering stage (*p* = 0.036 < 0.05; Table 1), between transgenic treatment and fertilizer-N level (*p* = 0.002 < 0.01) and between transgenic treatment and CO_2_ level (*p* = 0.005 < 0.01) on leaf sheath N% during heading stage (Table 1). Significant interactions among transgenic treatment, CO_2_, and fertilizer-N levels on leaf sheath N% (*p* = 0.037 < 0.05; Table 1) and C:N ratio (*p* = 0.004 < 0.01; Table 2) were also found during heading stage (Table 1 and Table 2). Compared with CK treatment, eCO_2_ significantly decreased N% (−22.92%) and increased C:N ratio (+33.86%) in leaf sheath of non-*Bt* rice during the tillering stage under high-N fertilizer, and those (N%: −11.32%; C:N ratio: +13.53%) of *Bt* rice during the tillering stage under low-N fertilizer (*p* < 0.05; Figure 1 and Figure 2). The eCO_2_ also significantly increased C:N ratio of *Bt* rice during heading stage under low-N fertilizer (+12.72%) and that of non-*Bt* rice during the tillering stage under low-N fertilizer (+36.62%; *p* < 0.05; Figure 2).

Under eTemp, T, fertilizer-N level, and transgenic treatment all significantly affected N% and C:N ratio in leaf sheath during the tillering and heading stages (*p* < 0.001 or 0.01), and there were significant interactions on N% between transgenic treatment and fertilizer-N during both tillering (*p* < 0.001) and heading (*p* = 0.047 < 0.05) stages, between transgenic treatment and T level during the tillering stage (*p* = 0.002 < 0.05), and among transgenic treatment, T and fertilizer-N level during the tillering (*p* = 0.014 < 0.05) and heading (*p* = 0.002 < 0.01) stages (Table 1). Moreover, there were significant interactions on C:N ratio between fertilizer-N and T level during the tillering stage (*p* = 0.027 < 0.05), and between transgenic treatment and fertilizer-N level during heading stage (*p* = 0.012 < 0.05; Table 2). Compared with CK treatment, eTemp significantly increased N% of non-*Bt* rice during the tillering (Low-N: +25.46%; High-N: +28.13%) and heading (Low-N: +22.08%; High-N: +8.70%) stages (*P* < 0.05; Figure 1), and significantly decreased C:N ratio of non-*Bt* rice during the tillering stage under both low-N (−22.35%) and high-N (−19.88%) fertilizer levels (*p* < 0.05; Figure 2); and eTemp significantly increased N% (Tillering: +18.87%; Heading: +16.33%) and decreased C:N ratio (Tillering: −13.71%; Heading: −14.84%) of *Bt* rice during the tillering and heading stages under low-N fertilizer (*p* < 0.05) and significantly decreased C:N ratio of *Bt* rice during heading stage under high-N fertilizer (−20.91%; *p* < 0.05; Figure 1 and Figure 2).

The Combined treatment only significantly affected N% (*p* = 0.011 < 0.05) and C:N ratio (*p* = 0.004 < 0.01) in leaf sheath during heading stage (Table 1 and Table 2) Transgenic treatment and fertilizer N significantly affected N% and C:N ratio in leaf sheath during the tillering and heading stages (*P* < 0.001 or *p* < 0.05; Table 1 and Table 2), and there were significant interactions between the combination of CO_2_ and T levels and fertilizer N on N% (*p* = 0.038 < 0.05) and C:N ratio (*p* < 0.001) during heading stage (Table 1 and Table 2). Compared with CK treatment, the Combined treatment significantly increased N% (*Bt* rice: +20.41%; non-*Bt* rice: +7.79%) and decreased C:N ratio (*Bt* rice: −16.39%; non-*Bt* rice: −7.32%) of *Bt* rice and non-*Bt* rice during heading stage under low-N fertilizer (*p* < 0.05; Figure 1 and Figure 2).

#### 2.1.2. N% and C:N Ratio of Rice Leaf

At eCO_2_ treatment, CO_2_, fertilizer-N levels, and transgenic treatment all significantly affected N% and C:N ratio in leaf of *Bt* and non-*Bt* rice during the tillering and heading stages (*p* < 0.001 or 0.01), except for the effect of transgenic treatment on N% during heading stage (Table 3 and Table 4). There were significant interactions on leaf N% between transgenic treatment and fertilizer-N level, and among CO_2_, transgenic treatment and fertilizer-N levels during the tillering and heading stages (*p* < 0.001 or *p* < 0.05), between transgenic treatment and CO_2_ level during the tillering stage, and between fertilizer-N and CO_2_ levels during heading stage (*p* < 0.001; Table 3). Significant interactions on C:N ratio in leaf were also found between transgenic treatment and CO_2_ level, between fertilizer-N and CO_2_ levels, and among CO_2_, fertilizer-N levels, and transgenic treatment during heading stage (*p* < 0.01 or 0.05; Table 4). Compared with CK, eCO_2_ significantly reduced N% and increased C:N ratio in leaf of *Bt* and non-*Bt* rice during the tillering and heading stages regardless of the N level (*p* < 0.05; Figure 3 and Figure 4).

Under eTemp, T, fertilizer-N level, and transgenic treatment all significantly affected N% and C:N ratio during the tillering and heading stages (*p* < 0.001), except for the effect of T on C:N ratio during the tillering stage (*p* = 0.36 > 0.05; Table 3 and Table 4). There were significant interactions on N% between transgenic treatment and T level, between transgenic treatment and fertilizer-N level, and between T and fertilizer-N levels during the tillering and heading stages (*p* < 0.001 or 0.05), except for the interaction between transgenic treatment and fertilizer-N level during the heading stage (Table 3). And significant interactions on C:N ratio in leaf were also found between transgenic treatment and T level during the tillering stage (*p* = 0.011 < 0.05), and between transgenic treatment and fertilizer-N level (*p* < 0.001), and among T, fertilizer-N level, and transgenic treatment during heading stage (*p* = 0.021 < 0.05; Table 4). Compared with CK, eTemp significantly increased N% of non-*Bt* rice during the tillering and heading stages under low- and high-N fertilizer (from +8.60% to 15.63%; *p* < 0.05), and significantly reduced C:N ratio of non-*Bt* rice during heading stage under high-N fertilizer (−7.16%; *p* < 0.05; Table 4); while eTemp only significantly increased N% (+8.86%) and decreased C:N ratio (−8.04%) of *Bt* rice during heading stage under low-N fertilizer (*p* < 0.05; Figure 3 and Figure 4).

The Combined treatment significantly affected N% (*p* < 0.001) and C:N ratio (*p* = 0.004 < 0.01) in leaf during heading stage (Table 3 and Table 4), and N% during the tillering stage (*p* = 0.029 < 0.05; Table 3), while transgenic treatment and fertilizer-N level significantly affected N% and C:N ratio in leaf during the tillering and heading stages (*p* < 0.001; Table 3 and Table 4). Furthermore, significant interactions on N% in leaf were also found during the tillering and heading stages between transgenic treatment and fertilizer-N level, between transgenic treatment and the combination of CO_2_ and T levels, and among transgenic treatment, fertilizer-N level and the combination of CO_2_ and T levels (*p* < 0.001 or 0.05; Table 3). Additionally, there were significant interactions on leaf C:N ratio between transgenic treatment and the combination of CO_2_ and T levels during the tillering stage (*p* = 0.006 < 0.01), between transgenic treatment and fertilizer-N level (*p* = 0.008 < 0.01), between transgenic treatment and the combination of CO_2_ and T levels (*p* = 0.043 < 0.05), and among transgenic treatment, fertilizer-N level, and the combination of CO_2_ and T levels during heading stage (*p* = 0.009 < 0.01; Table 4). Compared with CK, the Combined treatment significantly increased N% in leaf of non-*Bt* rice during the tillering and heading stages under low- and high-N fertilizer (from +6.25% to +10.17%; *p* < 0.05), but only significantly increased C:N ratio in leaf of non-*Bt* rice during heading stage under low-N fertilizer (7.84%; *p* < 0.05; Figure 3 and Figure 4); the Combined treatment only significantly decreased N% (−5.12% and −5.90%) and increased C:N ratio (+4.72% and +5.71%) in leaf of *Bt* rice during the tillering stage under low- and high-N fertilizer (*p* < 0.05; Figure 3 and Figure 4).

### 2.2. Bt Toxin Content in Leaf and Leaf Sheath of Bt Rice

**Figure 5:**
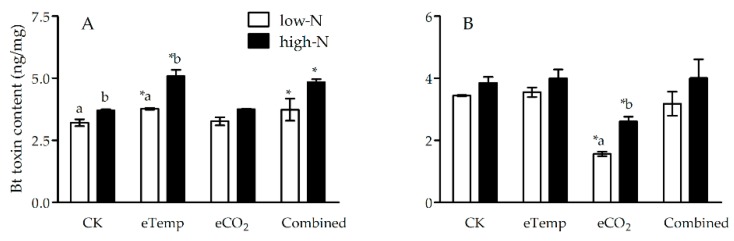
The *Bt* toxin content (ng/mg) in leaf sheath of transgenic *Bt* rice (cv. HH1) during the tillering (**A**) and heading (**B**) stages, grown with low- and high-N fertilizer under various CO_2_ and temperature (T) combination in open-top chambers. (Note: *, the lowercase (a, b) and uppercase letters (A, B) indicated significant difference in N% of *Bt* rice or non-*Bt* rice under eTemp, eCO_2_ or Combined treatment compared with CK at same fertilizer-N level, and between low-N and high-N for *Bt* rice or non-*Bt* rice grown under same levels of CO_2_ and T, and between *Bt* rice and non-*Bt* rice grown under same levels of CO_2_, T and N fertilizer by the Turkey-test at *p* < 0.05, respectively).

**Figure 6:**
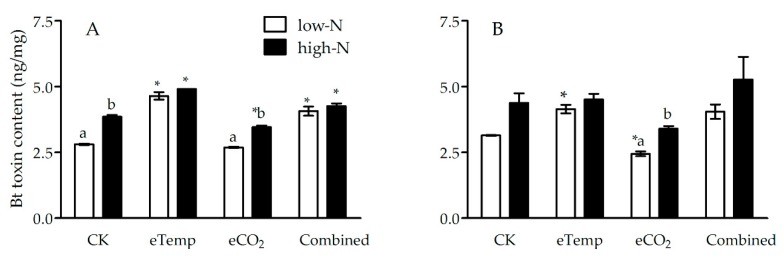
The *Bt* toxin content (ng/mg) in leaf of transgenic *Bt* rice (cv. HH1) during the tillering (**A**) and heading (**B**) stages, grown with low- and high-N fertilizer under various CO_2_ and temperature (T) combination in open-top chambers. (Note: *, the lowercase (a, b) and uppercase letters (A, B) indicated significant difference in N% of *Bt* rice or non-*Bt* rice under eTemp, eCO_2_ or Combined treatment compared with CK at same fertilizer-N level, and between low-N and high-N for *Bt* rice or non-*Bt* rice grown under same levels of CO_2_ and T, and between *Bt* rice and non-*Bt* rice grown under same levels of CO_2_, T and N fertilizer by the Turkey-test at *p* < 0.05, respectively).

Under eCO_2_ treatment, CO_2_ level significantly influenced *Bt*-toxin content in leaf sheath during heading stage (*p* < 0.001; Table 5) and leaf during the tillering (*p* < 0.001) and heading (*p* = 0.003 < 0.01) stages (Table 6), whereas fertilizer-N level significantly affected *Bt*-toxin content in both leaf sheath and leaf during the tillering and heading stages (*p* < 0.001 or 0.01; Table 5 and Table 6). Moreover, there were significant interactions between CO_2_ and fertilizer-N levels on *Bt*-toxin content in leaf sheath during heading stage (*p* = 0.038 < 0.05; Table 5) and leaf during the tillering stage (*p* = 0.017 < 0.05; Table 6). Compared with CK, eCO_2_ significantly decreased *Bt*-toxin content in leaf sheath during heading stage under low-N and high-N fertilizer levels (−54.65% and −32.38%) and leaf during the tillering stage under high-N fertilizer (−10.36%) and leaf during heading stage under low-N fertilizer (−22.22%; Figure 5 and Figure 6).

Under eTemp treatment, T and fertilizer-N levels significantly affected *Bt*-toxin content in leaf sheath during the tillering stage (*p* < 0.001), and there was a significant interaction between these two factors (*p* = 0.023 < 0.05; Table 5); T and fertilizer-N levels significantly affected *Bt*-toxin content in leaf during the tillering and heading stages (*p* < 0.001, 0.01 or 0.05), and there was a significant interaction between these two factors during the tillering stage (*p* = 0.008 < 0.01; Table 6). Compared with CK, eTemp significantly increased *Bt*-toxin content in leaf sheath (Low-N: −17.45%; High-N: −37.57%) and leaf (Low-N: −65.48%; High-N: −27.20%) during the tillering stage under low- and high-N fertilizer, and that (−31.75%) in leaf during heading stage under low-N fertilizer (*p* < 0.05; Figure 5 and Figure 6).

Moreover, the combination of CO_2_ and T levels and fertilizer-N levels only significantly affected *Bt*-toxin content in leaf sheath during the tillering stage (Table 5), and these two factors and their interaction all significantly affected *Bt*-toxin content in leaf during the tillering stage (*p* < 0.001, 0.01 or 0.05; Table 6), while fertilizer-N level only significantly influenced *Bt*-toxin content in leaf during heading stage (*p* = 0.036 < 0.05; Table 6). Compared with CK, the Combined treatment significantly increased *Bt*-toxin content in leaf sheath (Low-N: +16.51%; High-N: +30.81%) and leaf (Low-N: +44.84%; High-N: +10.36%) during the tillering stage under low- and high-N fertilizer (*p* < 0.05; Figure 5 and Figure 6).

Furthermore, compared with low-N fertilizer, high-N fertilizer significantly enhanced Bt-toxin content in leaf sheath and leaf of *Bt* rice during the tillering and heading stage grown under eCO_2_ (*p* < 0.05), except for those in leaf sheath during the tillering stage under low- and high-N fertilizer (*p* > 0.05; Figure 5 and Figure 6); high N level also significantly increased Bt-toxin content in leaf sheath and leaf of *Bt* rice during the tillering stage under CK treatment (*p* < 0.05), and significantly increased those in leaf sheath during the tillering stage under eTemp treatment (*p* < 0.05; Figure 5).

### 2.3. The Correlation Analysis of Foliar N%, C:N Ratios and Bt Toxin Production

The correlations between *Bt*-toxin content and N%, and C:N ratios in leaf and leaf sheath of *Bt* rice during the tillering and heading stages under low- and high-N fertilizer were analyzed (Supplementary Table S1). A significantly positive correlation between *Bt*-toxin content and N% (*R*^2^ = 1.000, *p* < 0.01) and a significantly negative correlation between *Bt*-toxin content and C:N ratio (*R*^2^ = −0.999, *p* < 0.05) in leaf sheath of *Bt* rice grown with low-N fertilizer under eTemp treatment (Supplementary Table S1) were observed. There was a significantly negative correlation between *Bt*-toxin content and N% in leaf of *Bt* rice grown with high-N fertilizer under CK treatment (*R*^2^ = −1.000, *p* < 0.01), and a significantly positive correlation between *Bt*-toxin content and N% in leaf of *Bt* rice grown with low-N fertilizer under CK treatment (*R*^2^ = 0.998, *p* < 0.05; Supplementary Table S1).

### 2.4. Effects of Various CO_2_ and Temperature Level Combinations on N. lugens Population Dynamics Fed on Bt and Non-Bt Rice Grown Under Low- and High-N Fertilizer

**Figure 7:**
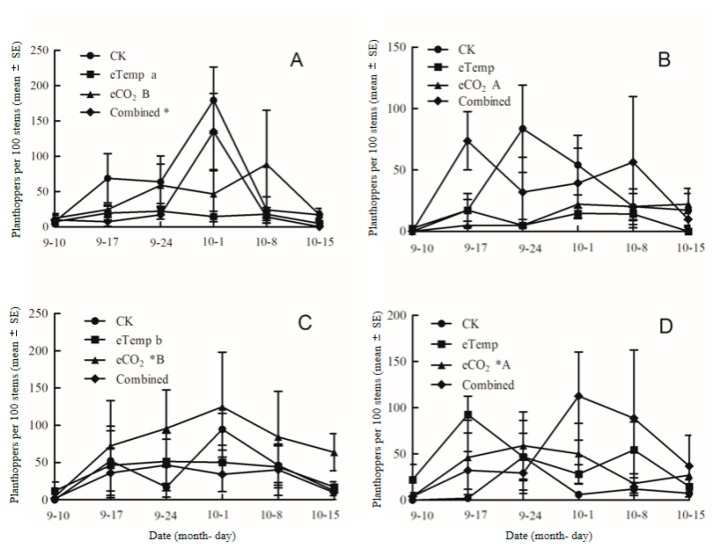
Population dynamics of brown planthopper, *Nilaparvata lugens* fed on transgenic *Bt* rice (cv. HH1) (**B**,**D**) and its parental isoline of non-*Bt* rice (cv. MH63) (**A**,**C**) grown under various CO_2_ and temperature (T) combinations with low-N (**A**,**B**) and high-N (**C**,**D**) fertilizer in open-top chambers from 10 Sept to 15 October 2016. (Note: *, the lowercase (a, b) and uppercase letters (A, B) indicated significant difference in N% of *Bt* rice or non-*Bt* rice under eTemp, eCO2 or Combined treatment compared with CK at same fertilizer-N level, and between low-N and high-N for *Bt* rice or non-*Bt* rice grown under same levels of CO_2_ and T, and between *Bt* rice and non-*Bt* rice grown under same levels of CO_2_, T and N fertilizer by the Turkey-test at *p* < 0.05, respectively).

At eCO_2_, transgenic treatment significantly affected the population dynamics of *N. lugens* fed on *Bt* rice versus non-*Bt* rice under low- and high-N fertilizer (*p* = 0.004 < 0.01), and there was a significant interaction between CO_2_ and fertilizer-N levels (*p* = 0.019 < 0.05), a significant interaction between T and fertilizer-N levels under ambient CO_2_ (*p* = 0.008 < 0.01; Table 7), and a marked interaction between transgenic treatment and the combination of CO_2_ and T levels (*p* = 0.081 < 0.10; Table 7). Compared with CK, eCO_2_ significantly enhanced the population dynamics of *N. lugens* fed on *Bt* and non-*Bt* rice under high-N fertilizer (*p* < 0.05; Figure 7C,D). Compared with CK, the Combined treatment significantly decreased the population dynamics of *N. lugens* fed on non-*Bt* rice under low-N fertilizer (*p* < 0.05; Figure 7A). Moreover, compared with low-N fertilizer, high-N fertilizer significantly enhanced the population abundance of *N. lugens* fed on non-*Bt* rice grown under eTemp treatment (*p* < 0.05; Figure 7A,C). Furthermore, compared with non-*Bt* rice, *Bt* rice significantly reduced the population abundance of *N. lugens* under eCO_2_ regardless of the N level (*p* < 0.05; Figure 7C,D).

## 3. Discussion

In accordance with many published literature, elevated CO_2_ significantly reduced the content of nitrogen and increased the C:N ratios in plants [30,31,32,33,34,35,36]. In this study, high temperature (T) had an opposite effect of elevated CO_2_ on the leaf nitrogen content (N%). When compared with the CK treatment (i.e., ambient CO_2_ and low T), the N% and C:N ratio of *Bt* rice and non-*Bt* rice were not significantly affected by the combined treatment of elevated CO_2_ and high T, except for leaf sheath during heading stage. This may be because of high T offsetting the effect of elevated CO_2_. Under elevated CO_2_ condition, the reduced N% may be attributed to the following reasons. First, some main carbon substances, such as starch, sugars and total nonstructural carbohydrates increased for plants grown under elevated CO_2_, which may result in a dilution effect and lower demand of nitrogen for plant growth [27]. Second, elevated CO_2_ reduced stomatal conductance of plants, which may result in weakened transpiration [37]. The transpiration offers the power for plants to transporting nitrogen from the root to the leaf sheath or the leaf [38], the weaken transpiration causes lower nitrogen content due to the difficulty in transporting nitrogen from root to leaf [39]. In addition, elevated CO_2_ can also increase the leaf temperature approximately 1–1.5 °C [40,41]. Moreover, Polley et al reported that high temperature increased evapotranspiration per unit of leaf area under all CO_2_ levels [42]. On the basis of summarizing a large number of results, Drake et al. found that elevated CO_2_ enhanced photosynthesis via increased Rubisco specificity for CO_2_ relative to O_2_, and the Rubisco specificity reduced as temperature increased, which implied there was an interaction effect among CO_2_, temperature and Rubisco activity [38]. In this study, the combined effects of elevated CO_2_ (even though its negative effect on N% and positive effect on C:N ratio) and high T (although its positive effect on N% and negative effect on C:N ratio) on N% and C:N ratio in leaf and leaf sheath of *Bt* rice and non-*Bt* rice were not significant regardless of low- and high-N fertilizer.

The ecological risks of elevated CO_2_ and temperature on *Bt* plants have received increasing attention since the worldwide adoption of *Bt* crops. In the current study, *Bt* rice grown in elevated CO_2_ produced lower level of *Bt* toxin than that grown under ambient CO_2_. The results were consistent with the previous studies that report the expression of exogenous-Bt toxin protein decreased under the elevated CO_2_ condition [21,43,44]. *Bt* rice grown at high temperature produces higher levels of *Bt* toxin than those plants grown at low temperature. The similar result were reported by Wu et al., who considered the expression of *cry1Ab* greatly influenced by temperature [45]. However, the *combined* treatment of elevated CO_2_ and high T only significantly increased the Bt-toxin content of *Bt* rice during the tillering stage.

General decreases in Bt-toxin and nitrogen contents and marked increases in C:N ratio have been found in *Bt* plants under elevated CO_2_ [46]. The N fertilizer was regarded as an attractive strategy to improve the C-N balance and the *Bt* toxin content in transgenic plants under elevated CO_2_ [47]_._ Our study indicated the high-N fertilizer increased the *Bt* toxin content of *Bt* rice when compared with low-N. In addition, there was no consistent relationship between the *Bt*-toxin content and N% or C:N ratio in leaf sheath and leaf of *Bt* rice grown under various CO_2_ and T combination with N-fertilizer supply, except for a significantly positive correlation between *Bt*-toxin content and N%, and a significantly negative correlation between *Bt*-toxin content and C:N ratio in leaf sheath of *Bt* rice grown with low-N fertilizer at high T and under ambient CO_2_ during the tillering stage.

In the current study, the abundance of *N. lugens* fed on *Bt* rice was significantly lower than that fed on non-*Bt* rice. Similarly, surveys of experimental fields have indicated that *N. lugens* migrate from *Bt* rice fields to adjacent non-*Bt* rice fields, resulting in low densities in *Bt* rice [11,48,49]. Compared with ambient CO_2_ at low T, elevated CO_2_ significantly enhanced the population dynamics of *N. lugens* fed on *Bt* rice and non-*Bt* rice under high-N fertilizer. The results indicated that the rising of the atmosphere CO_2_ could increase the risk of the *N. lugens* outbreaks. Similarly, Bezemer et al. found that the long-term exposure to elevated CO_2_ increased the abundance of *Myzus persicase* [50]. Massad and Dyer also found that elevated CO_2_ increased herbivory on the basis of a meta-analysis [28]. However, the insects vary in their responses to plants grown in elevated CO_2_. Hughes and Bazzaz found that the responses of aphid populations to elevated CO_2_ was species-specific with *Myzus persicae* increased, *Acyrthosiphon pisum* decreased, and the other species (*Aphis nerii*, *Aphis oenotherae* and *Aulacorthum solani*) unaffected [51]. At high temperature, the abundance of *N. lugens* fed on non-*Bt* rice were significantly increased under high-N fertilizer. One possible explanation for the increase is that high temperature improved N% and reduced the C:N ratio, carried out the role of nitrogen, which modified the plant nutrition and reduced resistance of non-*Bt* rice against *N. lugens* [52,53]. Additionally, Wan et al. reported that different planthopper species (*N. lugens*, *Laodelphax striatellus* and *Sogatella fucifera*) displayed species-specific responses to elevated CO_2_ and temperature [12]. Moreover, the negative effect of elevated CO_2_ and the positive effect of high temperature on insect performance and plant quality resulted in the combined interaction of elevated (CO_2_) and high temperature that did not significantly influence the population dynamics of *N. lugens*.

Under future predicted climate changes, it is particularly important to understand the direct and indirect effects of multiple environmental factors on insect herbivores. In the current study, the combined treatment which incorporated the positive effect of high temperature and negative effect of elevated CO_2_ together, to some extent, did not influence the N%, C:N ratio of *Bt* and non-*Bt* rice or *Bt* toxin production of *Bt* rice. Similarly, the abundance of *N. lugens* was not affected by the Combined treatment of elevated CO_2_ and high temperature. Our findings show that the plant of *Bt* rice expressing *cry1Ab/1Ac* would not trigger *N. lugens* outbreak under the higher temperature and higher concentrations of carbon dioxide in the future. Hence, we conclude that the plant of *Bt* rice will not only control the target lepidopteran pest effectively, but it can also control the abundance of non-target planthopper.

## 4. Materials and Methods

### 4.1. Open-Top Chambers

The experiment was conducted in 12 open-top chambers (ab., OTCs) in Ningjin County, Shandong Province of China (37º38′ 30.7′′ N, 116º51′ 11.0′′ E). The OTC is 2.5 m in height × 3.2 m in diameter. The open top chamber wall is composed of transparent glass, and the top of the chamber is composed of mesh. Two levels of CO_2_ concentration, ambient level (i.e., 375 µL/L) and elevated level (i.e., 650 µL/L, representing the predicted level in about 100 years) were continuously applied. In addition, two temperature (ab., T) levels, low temperature (i.e., ambient; ab., Low-T) and high temperature (i.e., ambient + 0.6 °C; ab., High-T) were applied continuously from July 10 to October 30 in 2016. These 12 OTCs were divided into four CO_2_ × T treatments, i.e., CK (ambient CO_2_ and Low-T), *e*Temp (ambient CO_2_ and High-T), *e*CO_2_ (elevated CO_2_ and Low-T) and Combined treatment (elevated CO_2_ and High-T). Three OTCs were used for each CO_2_ × T treatment.

During the experiment, the CO_2_ was continuously provided with tank CO_2_ gas (99% purity) and adjusted by using an infrared CO_2_ analyzer (Ventostat 8102; Telaire Company, Goleta, CA, USA), and temperature levels were monitored continuously by using an automatic temperature analysis system (U23-001, HOBO Pro V2 Temp/RH Data Logger; MicroDAQ.com, Ltd, Contoocook, NH, USA). Moreover, each OTC was divided into four similar units by plastic netting (mesh size: 0.15 mm × 0.15 mm) to prevent brown planthoppers escaping and mixing.

### 4.2. Rice Cultivars

The *Bt* rice expressing *Cry1Ab/Ac* genes (cv., Huahui-1; ab., HH1) and its corresponding parental isoline, Minghui 63 (ab., MH63) were used in this experiment. The seeds of the above two rice cultivars were provided by Prof. Yongjun Lin from Huazhong Agricultural University, Wuhan, Hubei Province of China. The rice seeds were cultivated in blue plastic bucket (26 cm in diameter × 35 cm in height) filled with moisture soil in 23 June 2016. The soil was then sampled and triturated to analyze its chemical composition (Institute of Soil Science and Chinese Academy of Science). Soil pH was 7.3, organic matter 12.3%, available N 215.2 mg/kg (hydrolic N, 1 m NaOH hydrolysis), available P 145.8 mg/kg (0.5 m NaHCO_3_ extraction) and available K 105.9 mg/kg (1 m CH_3_COONH_4_ extraction).

Two units in each OTC were used for *Bt* rice (cv. HH1) and its parental isoline (cv. MH63) grown with low-N (i.e., 75 kg/hm^2^) and high-N (i.e., 300 kg/hm^2^) fertilizers, respectively. Five buckets of per rice cultivar for each N fertilizer treatment were placed randomly in each OTC. According to the field planting density, five strains of rice were confirmed in each bucket when the seedlings were in the three-leaf stage. The nitrogen fertilizer was supplied with the addition of carbamide (analytical grade), which was divided into three parts, including of seedling, tillering, and heading fertilizers with the ratio of 4:3:3. These buckets were watered regularly to ensure sufficient moisture and no pesticide was used throughout the experiment. Moreover, the tiller number was recorded on the heading stage. In addition, the rice samples (leaf and leaf sheath) were got during the tillering stage and the heading stage respectively to analyze the foliar contents of nitrogen, carbon, and Bt toxin protein.

### 4.3. Rice Planthopper

The brown planthopper, *N*. *lugens* were collected from the Jiangpu paddyfield in Nanjing, Jiangsu Province of China on 1 July 2015, and were continuously reared on the susceptible rice cultivar TN1 (provided by the International Rice Research Institute, Philippines) in greenhouses (Photoperiod: 16L:8D; Temperature: 26.0 ± 1.0 °C; RH: 90 ± 10%) until used for inoculation treatments on 26 August 2016.

### 4.4. Rice Nitrogen, Carbon, and Bt Toxin Content

The foliar contents of nitrogen and carbon was assayed by using the CNH analyzer (Model: ANCA-nt, Europa Elemental Instruments,Hanau, Germany) of the Institute of Soil Science, Chinese Academy of Sciences, Nanjing, Jiangsu Province of China. Moreover, the foliar content of *Bt* toxin protein were measured in our laboratory by using a commercially-available ELISAs (Agdia, Elkhart, IN, USA).

### 4.5. Insect Inoculation and Population Abundance

On 26 August, ten pairs of newly emerged (<24 h after emergence) adults of macropterous females and males were randomly collected from the rice planthopper stocks for inoculation in each bucket of *Bt* rice (cv. HH1) and its parental isoline (cv. MH63) in each OTC, respectively. After 15 days of continuous rearing, three buckets of each N-fertilizer treatment for each rice cultivar were randomly selected from each OTC, and the nymphs and adults of *N. lugens* were counted every 7 days from September 10 to October 15. Population abundances were converted to the numbers of *N. lugens* per 100 stems.

### 4.6. Data Analysis

All data were analyzed by using the statistical software SPSS 19.0 (2015, SPSS Institute, Chicago, IL, USA). In order to make clear the respective effects of CO_2_ and T levels, and their combination effects on N% and C:N ratio in leaf sheath and leaf of *Bt* and non-*Bt* rice grown with low- and high-N fertilizer during the tillering and heading stages, three-way analysis of variances (ab., ANOVAs) were used to analyze the effects of CO_2_ (elevated CO_2_ versus ambient CO_2_) or T (high T versus low T) or their combination (elevated CO_2_ and high T versus ambient CO_2_ and low T), fertilizer-N level (High-N versus Low-N), transgenic treatment (*Bt* rice versus non-*Bt* rice) and their bi- and tri-interactions on the measured nutrients’ indexes of *Bt* and non-*Bt* rice during the sampling period, respectively. Moreover, two-way ANOVAs were also used to analyze the respective effects of CO_2_ /T level or their combination, fertilizer-N level, and their interactions on *Bt*-toxin content in leaf sheath and leaf of *Bt* rice during the tillering and heading stages, respectively. Furthermore, the Pearson’s correlation analysis was used to analyze the significant difference between the foliar N% and C:N ratio and *Bt* toxin production in leaf sheath and leaf of *Bt* rice grown with low- and high-N fertilizer during the tillering and heading stages. Moreover, in order to make clear the respective effects of CO_2_ and T levels, and their combination effects on the population dynamics of *N. lugens* fed on *Bt* and non-*Bt* rice grown with low- and high-N fertilizer from 10 Sept to 15 Oct in 2016, three-way repeated-measures ANOVAs (every 7 days sampling dates as the repeated measures) were also used to analyze the effects of CO_2_ /T level or their combination, fertilizer-N level, transgenic treatment, and their bi- and tri-interactions on the population abundances of *N. lugens* during the sampling period. Furthermore, the Pearson analysis was further used to analyze the relationships between Bt-toxin content and N% or C:N ratios in leaf and leaf sheath of *Bt* rice during the tillering and heading stages under low- and high-N fertilizer, respectively. And means of different treatments were separated by using the Tukey test to examine significant difference at *p* < 0.05. Abundance data were log transformed to normalize prior to analysis.

## Figures and Tables

**Table 1:** df, *F*-values and *p*-values derived from the three-way ANOVAs on nitrogen content (N%) in leaf sheath of transgenic *Bt* rice (cv. HH1) and its parental isoline of non-*Bt* rice (cv. MH63) during the tillering and heading stages grown under variousCO_2_ and temperature (T) in combinations with low- and high-N fertilizer in open-top chambers (*n* = 3).

Factors	*e*CO_2_		*e*Temp		Combined	
	Tillering	Heading	Tillering	Heading	Tillering	Heading
*Bt*	1, 1.75, 0.205	1, 196.40, <0.001 ***	1, 43.09, <0.001 ***	1, 292.83, <0.001 ***	1, 7.00, 0.018 *	1, 383.71, <0.001 ***
N	1, 156.22, 0.001 ***	1, 85.56, <0.001 ***	1, 401.14, <0.001 ***	1, 72.15, <0.001 ***	1, 389.96, <0.001 ***	1, 43.39, <0.001 ***
*e*Temp	-	-	1, 46.43, <0.001 ***	1, 71.98, <0.001 ***	-	-
*e*CO_2_	1, 13.84, 0.002 **	1, 23.02, <0.001 ***	-	-	-	-
Combined	-	-	-	-	1, 1.47, 0.242	1, 8.39, 0.011 *
*Bt* × N	1, 1.59, 0.695	1, 13.82, 0.002^**^	1, 19.98, 0.001 ***	1, 4.64, 0.047^*^	1, 4.43, 0.051	1, 2.78, 0.115
*Bt* × *e*Temp	-	-	1, 13.71, 0.002 **	1, 0.91, 0.353	-	-
N × *e*Temp	-	-	1, 0.47, 0.503	1, 0.75, 0.399	-	-
*Bt* × *e*CO_2_	1, 0.75, 0.399	1, 10.65, 0.005 **	-	-	-	-
N × *e*CO_2_	1, 5.25, 0.036 *	1, 3.83, 0.068	-	-	-	-
*Bt* × Combined	-	-	-	-	1, 0.06, 0.803	1, 1.90, 0.187
N × Combined	-	-	-	-	1, 0.02, 0.884	1, 5.13, 0.038 *
*Bt* × N × *e*Temp	-	-	1, 7.54, 0.014^*^	1, 13.40, 0.002 **	-	-
*Bt* × N × *e*CO_2_	1, 2.95, 0.105	1, 5.19, 0.037 *	-	-	-	-
*Bt* × N × Combined	-	-	-	-	1, 0.13, 0.725	1, 0.01, 0.937

**Note:***e*CO_2_–CO_2_ levels (elevated versus ambient) at low T; *e*Temp—T levels (high versus low) under ambient CO_2_; Combined—combinations of CO_2_ and T (elevated CO_2_ and high-T versus ambient CO_2_ and low T). * *p* < 0.05, ** *p* < 0.01, *** *p* < 0.001. Same explanation of variables for Tables 2–7.

**Table 2:** df, *F*-values and *p*-values derived from the three-way ANOVAs on C:N ratio in leaf sheath of transgenic *Bt* rice (cv. HH1) and its parental isoline of non-*Bt* rice (cv. MH63) during the tillering and heading stages grown under various CO_2_ and temperature (T) in combinations with low- and high-N fertilizer in open-top chambers (*n* = 3).

Factors	*e*CO_2_		*e*Temp		Combined	
	Tillering	Heading	Tillering	Heading	Tillering	Heading
*Bt*	1, 2.67, 0.122	1, 146.60, <0.001 ***	1, 13.56, 0.002 **	1, 155.67, <0.001 ***	1, 3.17, 0.049 *	1, 366.80, <0.001 ***
N	1, 61.90, <0.001 ***	1, 64.44, <0.001 ***	1, 136.86, <0.001 ***	1, 36.78, <0.001 ***	1, 105.37, <0.001 ***	1, 49.27, <0.001 ***
*e*Temp	-	-	1, 13.99, 0.002 **	1, 20.21, <0.001 ***	-	-
*e*CO_2_	1, 8.76, 0.009 **	23.02/<0.001 ***	-	-	-	-
Combined	-	-	-	-	1, 0.01, 0.907	1, 11.35, 0.004 **
*Bt* × N	1,0.01, 0.9200	13.82/0.002 **	1, 2.54, 0.130	1, 8.43, 0.012 *	1, 1.29, 0.273	1, 1.44, 0.247
*Bt* × *e*Temp	-	-	1, 3.99, 0.063	1, 0.89, 0.359	-	-
N × *e*Temp	-	-	1, 5.89, 0.027 *	1, 1.33, 0.267	-	-
*Bt* × *e*CO_2_	1, 0.32, 0.582	10.65/0.005 **	-	-	-	-
N × *e*CO_2_	1, 1.25, 0.280	3.83/0.068	-	-	-	-
*Bt* × Combined	-	-	-	-	1, 0.20, 0.660	1, 4.46, 0.051
N × Combined	-	-	-	-	1, 0.81, 0.380	1, 19.47, <0.001 ***
*Bt* × N × *e*Temp	-	-	1, 0.24, 0.631 *	1, 0.40, 0.536	-	-
*Bt* × N × *e*CO_2_	1, 0.36, 0.556	1, 11.40, 0.004 **	-	-	-	-
*Bt* × N × Combined	-	-	-	-	1, 0.07, 0.796	1,5.75, 0.290

**Note:***e*CO_2_—CO_2_ levels (elevated versus ambient) at low T; *e*Temp—T levels (high versus low) under ambient CO_2_; Combined—combinations of CO_2_ and T (elevated CO_2_ and high-T versus ambient CO_2_ and low T). * *p* < 0.05, ** *p* < 0.01, *** *p* < 0.001.

**Table 3:** df, *F*-values and *P*-values derived from the three-way ANOVAs on nitrogen content (N%) in leaf of transgenic *Bt* rice (cv. HH1) and its parental isoline of non-*Bt* rice (cv. MH63) during the tillering and heading stages grown under various CO_2_ and temperature (T) in combinations with low- and high-N fertilizer in open-top chambers (*n* = 3).

Factors	*e*CO_2_		*e*Temp		Combined	
	Tillering	Heading	Tillering	Heading	Tillering	Heading
*Bt*	1, 69.76, <0.001 ***	1, 0.59, 0.455	1, 385.98, <0.001 ***	1, 42.36, <0.001 ***	1, 204.30, <0.001 ***	1, 60.14, <0.001 ***
N	1, 1734.54, <0.001 ***	1, 337.02, <0.001 ***	1, 3868.60, <0.001 ***	1, 145.59, <0.001 ***	1, 1568.68, <0.001 ***	1, 345.92, 0.001 ***
*e*Temp	-	-	1, 145.00, <0.001 ***	1, 138.62, 0.001 ***	-	-
*e*CO_2_	1, 151.35, <0.001 ***	1, 184.83, <0.001 ***	-	-	-	-
Combined	-	-	-	-	1, 5.76, 0.029 *	1, 32.19, <0.001 ***
*Bt* × N	1, 25.80, <0.001 ***	1, 39.63, <0.001 ***	1, 26.63, <0.001 ***	1, 0.05, 0.829	1, 20.01, <0.001 ***	1, 8.34, 0.011 *
*Bt* × *e*Temp	-	-	1, 217.78, <0.001 ***	1, 46.25, <0.001 ***	-	-
N × *e*Temp	-	-	1, 5.98, 0.026 *	1, 24.01, <0.001 ***	-	-
*Bt* × *e*CO_2_	1, 28.14, <0.001 ***	1, 0.28, 0.606	-	-	-	-
N × *e*CO_2_	1.83/0.194	1, 20.03, <0.001 ***	-	-	-	-
*Bt* × Combined	-	-	-	-	1, 128.76, <0.001 ***	1, 65.46, <0.001 ***
N × Combined	-	-	-	-	1, 0.43, 0.522	1, 0.76, 0.396
*Bt* × N × *e*Temp	-	-	1, 1.84, 0.193	1, 3.34, 0.086	-	-
*Bt* × N × *e*CO_2_	1, 7.34, 0.015 *	1, 63.53, <0.001 ***	-	-	-	-
*Bt* × N × Combined	-	-	-	-	1, 4.76, 0.044 *	1, 27.43, <0.001 ***

* *p* < 0.05, ** *p* < 0.01, *** *p* < 0.001.

**Table 4:** df, *F*-values and *P*-values derived from the three-way ANOVAs on C:N ratio in leaf of transgenic *Bt* rice (cv. HH1) and its parental isoline of non-*Bt* rice (cv. MH63) during the tillering and heading stages grown under various CO_2_ and temperature (T) in combinations with low- and high-N fertilizer in open-top chambers (*n* = 3).

Factors	*e*CO_2_		*e*Temp		Combined	
	Tillering	Heading	Tillering	Heading	Tillering	Heading
*Bt*	1, 9.85, 0.006 **	1, 80.90, <0.001 ***	1, 19.78, <0.001 ***	1, 48.78, <0.001 ***	1, 20.03, <0.001 ***	.1, 23.60, <0001 ***
N	1, 97.64, <0.001 ***	1, 44.25, <0.001 ***	1, 111.64 <0.001 ***	1, 32.86 <0.001 ***	1, 93.19, <0.001 ***	1, 20.47, <0.001 ***
*e*Temp	-	-	1, 0.89, 0.359	1, 26.25, <0.001^***^	-	-
*e*CO_2_	1, 22.13, <0.001 ***	1, 32.14, <0.001 ***	-	-	-	-
Combined	-	-	-	-	1, 0.20, 0.661	1, 11.06, 0.004 **
*Bt* × N	1, 1.36, 0.261	1, 0.81, 0.381	1, 0.61, 0.448	1, 25.92, <0.001 ***	1, 0.49, 0.494	1, 18.84, 0.008 **
*Bt* × *e*Temp	-	-	1, 8.47, 0.011 *	1, 0.14, 0.712	-	-
N × *e*Temp	-	-	1, 2.35, 0.145	1, 0.05, 0.832	-	-
*Bt* × *e*CO_2_	1, 3.80, 0.069	1, 15.77, 0.009 **	-	-	-	-
N × *e*CO_2_	1, 0.28, 0.605	1, 8.34, 0.011 *	-	-	-	-
*Bt* × Combined	-	-	-	-	1, 10.03, 0.006 **	1, 4.82, 0.043 *
N × Combined	-	-	-	-	1, 0.41, 0.530	1, 0.58, 0.456
*Bt* × N × *e*Temp	-	-	1, 0.40, 0.535	1, 6.56, 0.021 *	-	-
*Bt* × N × *e*CO_2_	1, 1.11, 0.308	1, 18.68, 0.008 **	-	-	-	-
*Bt* × N × Combined	-	-	-	-	1, 0.33, 0.571	1, 8.97, 0.009 **

* *p* < 0.05, ** *p* < 0.01, *** *p* < 0.001.

**Table 5:** df, *F*-values and *p*-values derived from the two-way ANOVAs on *Bt*-toxin content in leaf sheath of transgenic *Bt* rice (cv. HH1) during the tillering and heading stages grown under various CO_2_ and temperature (T) combinations with low- and high-N fertilizer in open-top chambers (*n* = 3).

Factors	*e*CO_2_		*e*Temp		Combined	
	Tillering	Heading	Tillering	Heading	Tillering	Heading
N	1, 19.85, 0.002 **	1, 33.44, <0.001 ***	1, 37.57, <0.001 ***	1, 5.18, 0.052	1, 10.88, 0.011 *	1, 2.78, 0.134
*e*Temp	-	-	1, 43.20, <0.001 ***	1, 0.45, 0.523	-	-
*e*CO_2_	1, 0.23, 0.644	1, 153.34, <0.001 ***	-	-	-	-
Combined	-	-	-	-	1, 11.74, 0.009 **	1, 0.02, 0.886
N × *e*Temp	-	-	1, 7.80, 0.023 *	1, 0.01, 0.934	-	-
N × *e*CO_2_	1, 0.01, 0.989	1, 6.20, 0.038 *	-	-	-	-
N × Combined	-	-	-	-	1, 1.60, 0.242	1, 0.30, 0.598

* *p* < 0.05, ** *p* < 0.01, *** *p* < 0.001.

**Table 6:** df, *F*-values and *p* values derived from the two-way ANOVAs on Bt-toxin content in leaf of transgenic Bt rice (cv. HH1) during the tillering and heading stages grown under various CO_2_ and temperature (T) combinations with low- and high-N fertilizer in open-top chambers (*n* = 3).

Factors	*e*CO_2_		*e*Temp		Combined	
	Tillering	Heading	Tillering	Heading	Tillering	Heading
N	1, 398.92, <0.001 ***	1, 31.49, 0.002 **	1, 68.34, <0.001 ***	1, 12.56, 0.008 **	1, 31.55, <0.009 **	1, 6.35, 0.036 *
*e*Temp	-	-	1, 334.08, <0.001 ***	1, 6.20, 0.037 *	-	-
*e*CO_2_	1, 31.69, <0.001 ***	1, 18.48, 0.003 **	-	-	-	-
Combined	-	-	-	-	1, 57.48, <0.001 ***	1, 3.36, 0.104
N × *e*Temp	-	-	1, 24.86, 0.002^**^	1, 3.67, 0.092	-	-
N × *e*CO_2_	1, 8.99, 0.017 *	1, 0.53, 0.486	-	-	-	-
N × Combined	-	-	-	-	1, 15.51, 0.004 **	1, 281.90, 0.985

* *p* < 0.05, ** *p* < 0.01, *** *p* < 0.001.

**Table 7:** df, *F*-values and *p* values derived from the three-way ANOVAs on population dynamics of *Nilaparvata lugens* fed on transgenic *Bt* rice (cv. HH1) and its parental isoline of non-*Bt* rice (cv. MH63) grown under various CO_2_ and temperature (T) combinations with low- and high-N fertilizer in open-top chambers from Sept 10 to Oct 15 in 2016 (*n* = 3).

Factors	*e*CO_2_	*e*Temp	Combined
*Bt*	1, 9.36, 0.004^**^	1, 2.13, 0.152	1, 0.34, 0.577
N	1, 0.09, 0.772	1, 0.15, 0.705	1, 0.44, 0.510
*e*Temp	-	1, 1.13, 0.292	-
*e*CO_2_	1, 0.26, 0.613	-	
Combined	-	-	1, 0.01, 0.951
*Bt* × N	1, 0.04, 0.851	1, 0.18, 0.68	1, 0.23, 0.642
*Bt* × *e*Temp	-	1, 2.22, 0.14	-
N × *e*Temp	-	1, 7.67, 0.008^**^	-
*Bt* × *e*CO_2_	1, 0.17, 0.695	-	-
N × *e*CO_2_	1, 5.96, 0.019^*^	-	-
*Bt* × Combined	-	-	1, 3.21, 0.081
N × Combined	-	-	1, 1. 56, 0.227
*Bt* × N × *e*Temp	-	1, 0.05, 0.826	-
*Bt* × N × *e*CO_2_	1, 0.13, 0.723	-	-
*Bt* × N × Combined	-	-	1, 0.11, 0.750

## Supplementary Materials

The following are available online at https://www.mdpi.com/2072-6651/11/5/261/s1, Table S1: Pearson correlation analysis of *Bt*-toxin content (ng/mg) with N% and C:N ratio in leaf sheath and leaf of *Bt* rice (cv. HH1) grown under various CO_2_ and temperature (T) combinations with low- and high-N fertilizer in open-top chambers (*R^2^*/*p* values)

## References

[1] Liu Q.S., Hallerman E., Peng Y.F., Li Y.H. (2016). Development of *Bt* rice and *Bt* maize in china and their efficacy in target pest control. Int. J. Mol. Sci..

[2] Wu K.M., Lu Y.H., Feng H.Q., Jiang Y.Y., Zhao J.Z. (2008). Suppression of cotton bollworm in multiple crops in china in areas with Bt toxin-containing cotton. Science.

[3] Hardee D.D., Bryan W.W. (1997). Influence of Bacillus thuringiensis-transgenic and nectariless cotton on insect populations with emphasis on the tarnished plant bug (heteroptera: *Miridae*). J. Econ. Entomol..

[4] Riddick E.W., Dively G., Barbosa P. (1998). Effect of a seed-mix deployment of Cry3A-transgenic and nontransgenic potato on the abundance of *Lebia grandis* (Coleoptera: Carabidae) and *Coleomegilla maculata* (Coleoptera: Coccinellidae). Ann. Entomol. Soc. Am..

[5] Wang Y.N., Zhang L., Li Y.H., Liu Y.M., Han L.Z., Zhu Z., Wang F., Peng Y.F. (1938). Expression of Cry1Ab protein in a marker-free transgenic Bt rice line and its efficacy in controlling a target pest, chilo suppressalis (Lepidoptera: Crambidae). Environ. Entomol..

[6] Chang X., Liu G.G., He K.L., Shen Z.C., Peng Y.F., Ye G.Y. (2013). Efficacy evaluation of two transgenic maize events expressing fused proteins to CrylAb-susceptible and -resistant *Ostrinia furnacalis* (Lepidoptera: Crambidae). J. Econ. Entomol..

[7] Chen H., Mang G., Zhang Q., Lin Y. (2008). Effect of transgenic *Bacillus thuringiensis* rice lines on mortality and feeding behavior of rice stem borers (Lepidoptera: Crambidae). J. Econ. Entomol..

[8] Wang Y.N., Ke K.Q., Li Y.H., Han L.Z., Liu Y.M., Hua H.X., Peng Y.F. (2014). Comparison of three transgenic Bt rice lines for insecticidal protein expression and resistance against a target pest, Chilo suppressalis (Lepidoptera: crambidae). Insect Sci..

[9] Wang B.J., Xu H.X., Zheng X.S., Fu Q., Lu Z.X. (2010). High temperature modifies resistance performances of rice varieties to brown planthopper, *Nilaparvata lugens* (Stâl). Rice Sci..

[10] Bernal C.C., Aguda R.M., Cohen M.B. (2010). Effect of rice lines transformed with Bacillus thuringiensis toxin genes on the brown planthopper and its predator *Cyrtorhinus lividipennis*. Entomol. Exp. Appl..

[11] Chen Y., Tian J.C., Wang W., Fang Q., Akhtar Z.R., Peng Y.F., Cui H., Guo Y.Y., Song Q.S., Ye G.Y. (2012). Bt rice expressing Cry1Ab does not stimulate an outbreak of its non-target herbivore, *Nilaparvata lugens*. Transgenic Res..

[12] Wan G., Dang Z., Wu G., Parajulee M.N., Ge F., Chen F. (2014). Single and fused transgenic *Bacillus thuringiensis* rice alter the species-specific responses of non-target planthoppers to elevated carbon dioxide and temperature. Pest Manag. Sci..

[13] Nowak R.S., Ellsworth D.S., Smith S.D. (2010). Functional responses of plants to elevated atmospheric CO_2_: Do photosynthetic and productivity data from face experiments support early predictions?. New Phytol..

[14] Wu G., Chen F.J., Ge F., Sun Y.C. (2007). Transgenic *Bacillus thuringiensis* (Bt) cotton (Gossypium hirsutum) allomone response to cotton aphid, *Aphis gossypii*, in a closed-dynamics CO_2_ chamber (CDCC). J. Plant Res..

[15] Wu G., Chen F.J., Xiao N.W., Ge F. (2011). Influences of elevated CO_2_ and pest damage on the allocation of plant defense compounds in Bt-transgenic cotton and enzymatic activity of cotton aphid. Insect Sci..

[16] Johnson S.N., Hartley S.E. (2018). Elevated carbon dioxide and warming impact silicon and phenolic-based defences differently in native and exotic grasses. Glob. Chang. Biol..

[17] Dai Y., Wang M.F., Jiang S.L., Zhang Y.F., Parajulee M.N., Chen F.J. (2018). Host-selection behavior and physiological mechanisms of the cotton aphid, *Aphis gossypii*, in response to rising atmospheric carbon dioxide levels. J. Insect Physiol..

[18] Qian L., He S., Liu X., Huang Z., Chen F.J., Gui F. (2018). Effect of elevated CO_2_ on the interaction between invasive thrips, *Frankliniella occidentalis*, and its host kidney bean, phaseolus vulgaris. Pest Manag. Sci..

[19] Murray T.J., Ellsworth D.S., Tissue D.T., Riegler M. (2013). Interactive direct and plant-mediated effects of elevated atmospheric [CO_2_] and temperature on a eucalypt-feeding insect herbivore. Glob. Chang. Biol..

[20] Zvereva E.L., Kozlov M.V. (2010). Consequences of simultaneous elevation of carbon dioxide and temperature for plant-herbivore interactions: A metaanalysis. Glob. Chang. Biol..

[21] Chen F.J., Wu G., Ge F., Parajulee M.N., Shrestha R.B. (2010). Effects of elevated CO_2_ and transgenic Bt cotton on plant chemistry, performance, and feeding of an insect herbivore, the cotton bollworm. Entomol. Exp. Appl..

[22] Johns C.V., Hughes L. (2010). Interactive effects of elevated CO_2_ and temperature on the leaf-miner dialectica scalariella zeller (Lepidoptera : Gracillariidae) in paterson’s curse, echium plantagineum (boraginaceae). Glob. Chang. Biol..

[23] Pritchard S.G., Rogers H.H., Prior S.A., Peterson C.M. (1999). Elevated CO_2_ and plant structure: A review. Glob. Chang. Biol..

[24] Way D.A., Oren R. (2010). Differential responses to changes in growth temperature between trees from different functional groups and biomes: A review and synthesis of data. Tree Physiol..

[25] Bale J.S., Masters G.J., Hodkinson I.D., Awmack C., Bezemer T.M., Brown V.K., Butterfield J., Buse A., Coulson J.C., Farrar J. (2010). Herbivory in global climate change research: Direct effects of rising temperature on insect herbivores. Glob. Chang. Biol..

[26] Hu G., Xie M.C., Lin Z.X., Xin D.Y., Huang C.Y., Chen W., Zhang X.X., Zhai B.P. (2010). Are outbreaks of nilaparvata lugens (Stâl) associated with global warming?. Environ. Entomol..

[27] Stiling P., Cornelissen T. (2010). How does elevated carbon dioxide (CO_2_) affect plant-herbivore interactions? A field experiment and meta-analysis of CO_2_-mediated changes on plant chemistry and herbivore performance. Glob. Chang. Biol..

[28] Massad T.J., Dyer L.A. (2010). A meta-analysis of the effects of global environmental change on plant-herbivore interactions. Arthropod-Plant Int..

[29] Jiang S.L., Liu T., Yu F., Li T., Parajulee M.N., Zhang L., Chen F.J. (2016). Feeding behavioral response of cotton aphid, *Aphis gossypii*, to elevated CO_2_: EPG test with leaf microstructure and leaf chemistry. Entomol. Exp. Appl..

[30] Lindroth R.L., Arteel G.E., Kinney K.K. (1995). Responses of three saturniid species to paper birch grown under enriched CO_2_ atmospheres. Funct. Ecol..

[31] Lincoln D.E. (1993). The influence of plant carbon-dioxide and nutrient supply on susceptibility to insect herbivores. Vegetatio.

[32] Coviella C.E., Stipanovic R.D., Trumble J.T. (2002). Plant allocation to defensive compounds: Interactions between elevated CO_2_ and nitrogen in transgenic cotton plants. J. Exp. Bot..

[33] Wilsey B.J. (1996). Plant responses to elevated atmospheric CO_2_ among terrestrial biomes. Oikos.

[34] Ceulemans R., Mousseau M. (1994). Tansley review NO.71 effects of elevated atmospheric CO_2_ on woody-plants. New Phytol..

[35] Cotrufo M.F., Ineson P., Scott A. (2010). Elevated CO_2_ reduces the nitrogen concentration of plant tissues. Glob. Chang. Biol..

[36] Zhang S., Fu W., Zhang Z., Fan Y., Liu T. (2017). Effects of elevated CO_2_ concentration and temperature on some physiological characteristics of cotton (*G**ossypium hirsutum* L.) leaves. Environ. Exp. Bot..

[37] Drake B.G., Gonzàlez-Meler M.A., Long S.P. (1997). More efficient plants: A consequence of rising atmospheric CO_2_?. Annu. Rev. Plant Boil..

[38] Conroy J., Hocking P. (2010). Nitrogen nutrition of C3 plants at elevated atmospheric CO_2_ concentrations. Physiol. Plant..

[39] Morison J.I. (1987). Intercellular CO_2_ concentration and stomatal response to CO_2_ james. Stomatal Funct..

[40] Katul G.G., Palmroth S., Oren R. (2010). Leaf stomatal responses to vapour pressure deficit under current and CO_2_-enriched atmosphere explained by the economics of gas exchange. Plant Cell Environ..

[41] Pinter P.J., Kimball B.A., Garcia R.L., Wall G.W., Hunsaker D.J., LaMorte R.L. (1996). 13-Free-air CO_2_ enrichment: Responses of cotton and wheat crops. Carbon Dioxide Terr. Ecosyst..

[42] Polley H., Johnson H., Fay P., Sanabria J. (2008). Initial response of evapotranspiration from tallgrass prairie vegetation to CO_2_ at subambient to elevated concentrations. Funct. Ecol..

[43] Chen M., Shelton A., Ye G.Y. (2010). Insect-resistant genetically modified rice in China: From research to commercialization. Annu. Rev. Entomol..

[44] Coviella C.E., Morgan D.J.W., Trumble J.T. (2000). Interactions of elevated CO_2_ and nitrogen fertilization: Effects on production of bacillus thuringiensis toxins in transgenic plants. Environ. Entomol..

[45] Wu G., Cui H., Ye G., Xia Y., Sardana R., Cheng X., Li Y., Altosaar I., Shu Q. (2002). Inheritance and expression of the Cry1Ab gene in Bt (*Bacillus thuringiensis*) transgenic rice. Theor. Appl. Genet..

[46] Chen F.J., Ge F. (2005). and Parajulee, M.N. Impact of elevated CO_2_ on Tri-Trophic interaction of *Gossypium hirsutum*, *Aphis gossypii*, and Leis axyridis. Environ. Entomol..

[47] Jiang S.L., Lu Y.Q., Dai Y., Qian L., Muhammad A.B., Li T., Wan G.J., Parrajulee M.N., Chen F.J. (2017). Impacts of elevated CO_2_ on exogenous Bacillus thuringiensis toxins and transgene expression in transgenic rice under different levels of nitrogen. Sci. Rep..

[48] Chen M., Liu Z.C., Ye G.Y., Shen Z.C., Hu C., Peng Y.F., Altosaar I., Shelton A.M. (2007). Impacts of transgenic cry1Ab rice on non-target planthoppers and their main predator Cyrtorhinus lividipennis (Hemiptera: Miridae)—A case study of the compatibility of Bt rice with biological control. Biol. Control.

[49] Chen M., Ye G., Hu C., Datta S. (2002). Effects of transgenic Bt indica rice on the feeding and oviposition behavior of the brown planthopper, *Nilaparvata lugens*. Acta Phytophylacica Sin..

[50] Bezemer T.M., Jones T.H., Knight K.J. (1998). Long-term effects of elevated CO_2_ and temperature on populations of the peach potato aphid myzus persicae and its parasitoid aphidius matricariae. Oecologia.

[51] Hughes L., Bazzaz F.A. (2010). Effects of elevated CO_2_ on five plant-aphid interactions. Entomol. Exp. Appl..

[52] Cisneros J.J., Godfrey L.D. (2001). Midseason pest status of the cotton aphid (Homoptera: Aphididae) in california cottonis nitrogen a key factor?. Environ. Entomol..

[53] Noldus L., Rumei X., Lenteren J.V. (1986). The parasite-host relationship between encarsia formosa gahan (Hymenoptera, Aphelinidae) and trialeurodes vaporariorum (Westwood) (Homoptera, Aleyrodidae). XVIII. Between-plant movement of adult greenhouse whiteflies. J. Appl. Entomol..

