# Supplementary material for: Interactive Effects of [CO2] and Temperature on Plant Chemistry of Transgenic Bt Rice and Population Dynamics of a Non-Target Planthopper, Nilaparvata lugens (Stål) under Different Levels of Soil Nitrogen
Source: Toxins (Basel). 2019 May 8;11(5):261. doi: 10.3390/toxins11050261 (PMC6562872; doi:10.3390/toxins11050261)
Supplement: Supplementary file 1 [file toxins-11-00261-s001.pdf]

Supplementary Materials: Interactive Effects of [CO_2] and Temperature on Plant Chemistry of Transgenic *Bt* Rice and Population Dynamic of a Non-Target Planthopper, *Nilaparvata lugens* (Stål) under Different Levels of Soil Nitrogen

Yanmin Liu, Zhihao Dang, Yanhui Wang, Megha N. Parajulee and Fajun Chen

Table S1. Pearson correlation analysis of *Bt*-toxin content (ng/mg) with N% and C:N ratio in leaf sheath and leaf of *Bt* rice (cv. HH1) grown under various CO_2 and temperature (T) combinations with low- and high-N fertilizer in open-top chambers (R^2/p values).

Rice stage	Fertilizer-N level	Treatment	Leaf Sheath		Leaf	
			N (%)	C:N Ratio	N (%)	C:N ratio
Tillering	Low N	CK	−0.124	−0.241	−0.387	0.384
		*e*Temp	1.000 **	−0.999 *	−0.194	−0.391
		eCO_2	−0.853	−0.307	−0.178	0.075
		Combined	−0.304	−0.701	−0.883	0.883
	High N	CK	0.619	0.590	−1.000 **	0.993
		*e*Temp	0.994	−0.981	0.227	−0.286
		eCO_2	0.983	−0.966	−0.888	0.888
		Combined	0.270	−0.270	−0.326	0.270
Heading	Low N	CK	−0.711	0.585	0.998 *	−0.993
		*e*Temp	−0.791	0.798	−0.830	0.839
		eCO_2	0.680	−0.796	−0.400	−0.348
		Combined	0.839	−0.835	0.988	−0.988
	High N	CK	−0.167	−0.008	−0.511	0.389
		*e*Temp	−0.952	0.938	0.718	−0.870
		eCO_2	0.906	−0.912	−0.928	0.982
		Combined	−0.956	0.953	0.988	−0.993

Note: * $p < 0.05$; ** $p < 0.01$.
